# Antioxidant Activity, LC-MS/MS Identification and *In Silico* Analysis of the Ethanol Extract of Beneng Taro Leaves (*Xanthosoma undipes* K. Koch) Grown in Three Different Locations

**DOI:** 10.21315/tlsr2026.37.1.2

**Published:** 2026-03-31

**Authors:** Diana Widiastuti, Rudi Dwi Hariyanto, Siska Elisahbet Sinaga, Wira Eka Putra, Sustiprijatno, Ifa Manzila, M Ace Suhendar, Triastinurmiatiningsih, Surya Diantina

**Affiliations:** 1 Department of Chemistry, Faculty of Mathematics and Natural Science, Universitas Pakuan, 16129 West Java, Indonesia; 2 Faculty of Agriculture, Yamagata University, 997-0037 Tsuruoka, Japan; 3 Biotechnology Study Program, Department of Applied Sciences, Faculty of Mathematics and Natural Sciences, Universitas Negeri Malang, 65145 East Java, Indonesia; 4 Research Center for Applied Botany, National Research and Innovation Agency, Jl. Raya Jakarta Bogor KM. 46. Cibinong, Bogor, 16911 West Java, Indonesia; 5 Research Center for Horticulture, National Research and Innovation Agency, Jl. Raya Jakarta Bogor KM. 46. Cibinong, Bogor, 16911 West Java, Indonesia; 6 Research Center for Food Crop, National Research and Innovation Agency, Jl. Raya Jakarta Bogor KM. 46. Cibinong, Bogor, 16911 West Java, Indonesia; 7 Department of Biology, Faculty of Mathematics and Natural Science, Universitas Pakuan, 16129 West Java, Indonesia

**Keywords:** Active Compounds, Antioxidant, Beneng Taro Leaves, LC-MS/MS, Planting Location

## Abstract

*Xanthosoma undipes* K. Koch (Beneng taro) leaves are rich in bioactive compounds with reported antioxidant and anticancer potential. This study evaluated the antioxidant activity of ethanol extracts from Beneng taro leaves cultivated in three locations, identified active compounds using liquid chromatography–tandem mass spectrometry (LC-MS/MS), and assessed their anticancer potential through in silico analysis. Extraction was performed with 96% ethanol, followed by phytochemical screening, quantification of total flavonoids and phenolics, and antioxidant evaluation using the DPPH assay. LC-MS/MS identified several bioactive phenolic and flavonoid compounds, including quercetin, isorhamnetin, hispidulin, oleocanthal and cyclovalone, as key contributors to antioxidant activity. Leaves from high-altitude Cisarua (TB-1) had the highest flavonoid (94.49 ± 1.61 mg QE/g) and phenolic (97.35 ± 1.74 mg GAE/g) contents, with the strongest antioxidant activity (IC_50_ = 42.96 μg/mL). Drug-likeness screening indicated favourable pharmacokinetic properties for several compounds. Molecular docking revealed strong binding affinities of quercetin, isorhamnetin, hispidulin, cochliophilin A, cyclovalone and oleocanthal to Kirsten rat sarcoma viral oncogene homolog (KRAS), a key oncogenic protein regulating cell growth, division and mutation, suggesting potential anticancer effects. These findings indicate that cultivation at higher altitudes enhances bioactive compound levels and antioxidant potential. Future studies should isolate active compounds, validate their effects i*n vivo*, optimise cultivation practices, and explore their development into functional foods or nutraceuticals.

**HIGHLIGHTS**

- Growing locations significantly affect the phytochemical composition and antioxidant activity of Beneng taro leaves, with high-altitude Cisarua (TB-1) exhibiting the highest flavonoids and phenolics content.
- Molecular docking analysis showed that quercetin, isorhamnetin and cochliophilin A exhibited the strongest binding affinity against KRAS, indicating potential anticancer properties.
- The study suggests that bioactive compounds from Beneng taro leaves could be developed into functional foods or pharmaceutical agents, warranting further *in vivo* and clinical analysis.

## INTRODUCTION

The use of herbal medicine is increasing globally, as more people turn to natural ingredients for treatment and health maintenance (Widiastuti *et al*. 2024). One medicinal plant commonly used is taro, a perennial food crop belonging to the Araceae family (Das *et al*. 2022). Traditionally, taro has been valued for its nutrient-rich tubers, while other parts of the plant, such as leaves and stems, are often underused or discarded (Khan *et al*. 2014). This practice not only overlooks the potential use of valuable plant resources but also poses environmental challenges. For instance, the excessive disposal of Beneng taro leaves into water bodies can disrupt aquatic ecosystems (Mitharwal *et al*. 2022). Recent investigations have highlighted the untapped potential of leaves. According to Shah *et al*. (2022), Beneng taro stalks and leaves were safe and considered effective sources of antioxidants, suggesting that they could be explored for great potential for further use.

Beneng taro leaves are known for their various therapeutic properties, including anticancer, antioxidant, antihyperlipidemic, anti-inflammatory, antihypertensive, antidiabetic, antimicrobial and antibacterial activities. Importantly, leaves contain a range of beneficial compounds such as flavonoids, alkaloids, tannins, triterpenoids, steroids and saponins (Lebot & Legendre 2015). These compounds have been widely shown to have potent antioxidant and anti-inflammatory properties, contributing significantly to the therapeutic potential of Beneng taro leaves. Despite such promising bioactivity, the full potential of the leaves remains largely unexplored, warranting further investigations to better understand and harness their functional properties. In line with this, a study conducted by Safwa *et al*. (2023) successfully isolated flavonol-type flavonoids from the stalk skin of taro leaves belonging to the same genus, *Xanthosoma*, which showed significant antioxidant potential.

Comparative studies in other *Xanthosoma* species, such as *X. sagittifolium* and *X. taioba*, have also reported variations in flavonoid content, phenolic profiles, and bioactivities depending on environmental conditions, suggesting a strong genotype–environment interaction that may apply to *X. undipes* as well (Rodríguez-Marin *et al*. 2019; De Jesus Benevides *et al*. 2022). These findings support the rationale for location-based comparisons in phytochemical studies.

The composition and concentration of secondary metabolites in the plant is influenced by several internal and external factors. Internal factors include genetics, plant conditions and age, while external factors involve light, temperature, soil nutrients and altitude. However, cultivation locations are particularly significant as they affect plant growth and development. This makes the metabolic processes in the plant varies, leading to differences in the bioactive compounds produced at different altitudes (Mayanti *et al*. 2022; Mulyani *et al*. 2023; Widiastuti *et al*. 2024).

The therapeutic potential of Beneng taro leaves in cancer treatment has been shown through their inhibitory effects on various cancer cell lines, including murine breast cancer, human breast cancer, human hepatoma HepG2 cells, human glioblastoma and others (Pereira *et al*. 2021). The results show the potential of the plant as a natural source of bioactive compounds for cancer prevention and therapy. However, there have been limited investigations on the anticancer properties of taro from the species *Xanthosoma undipes*.

To address this gap, the current study conducted an *in silico* analysis to predict the anticancer potential of *X. undipes* by targeting the Kirsten rat sarcoma viral oncogene homolog (KRAS) protein. KRAS is a well-established oncogenic driver in multiple human cancers. It regulates pathways critical for cell proliferation and survival, and mutations in KRAS are associated with poor prognosis and resistance to targeted therapies (Yang *et al*. 2024). While KRAS has been widely studied in synthetic drug development, relatively few natural product-based inhibitors have been explored. Investigating Beneng taro-derived compounds against KRAS provides a novel angle in natural anticancer research.

Based on the above discussion, this study aims to evaluate the antioxidant activity of the ethanol extract from Beneng taro leaves grown in three different locations. It also aims to identify active compounds present in the leaves using LC-MS/MS and assess the extract’s anticancer potential through *in silico* analysis. The total flavonoids and phenolics contents were measured to examine the effect of cultivation locations on metabolite composition. Additionally, antioxidant activity was evaluated through *in vitro* analysis to identify the extract with the highest activity, providing a basis for future investigations on the potential applications of Beneng taro leaves extract. The *in silico* analysis offers insights into optimising specific compounds for development as potential anticancer drug candidates.

## MATERIALS AND METHODS

### Sample Preparation

This study collected samples from three different cultivation areas: Kp. Cisarua Dalam (Cisarua Sub-district, Bogor Regency, labelled as TB-1), Cimanggu (Tanah Sereal Sub-district, Bogor City, labelled as TB-2), and Bogor (Bogor City, labelled as TB-3). Species identification was conducted at the Herbarium Depokensis, Biota Collection Room, University of Indonesia, confirming that the plant samples were classified as Beneng taro leaves (*Xanthosoma undipes* K. Koch) with identification number JI23-P-189. Subsequently, the leaves were sorted and washed under tap water to remove adhering impurities. Beneng taro leaves were cut into smaller pieces and dried in an oven at 50°C for 4 h before being grounded to obtain fine simplicia powder.

### Moisture Content Determination

About 3 g of the simplicia powder was dried in an oven at 105°C for 3 h. Subsequently, it was cooled in a desiccator for 15 min, and the weight was recorded. The weight was recorded and repeated until a constant weight was obtained.

### Sample Extraction

A total of 50 g of taro leaves simplicia was soaked in 500 mL of 96% ethanol for 3 × 24 h in a sealed container at room temperature with occasional stirring. The extract was filtered to collect the filtrate and concentrated using a rotary evaporator at 40°C–55°C. The thick extract obtained was weighed to calculate the extraction yield (Widiastuti *et al*. 2023).

### Phytochemical Screening

Flavonoid testing was conducted by placing approximately 1 mL of the crude taro leaves extract into a test tube, followed by the addition of magnesium powder and a few drops of concentrated hydrochloric acid (HCl). Another test involved adding a few drops of 10% sodium hydroxide (NaOH) to the extract in a separate test tube. A positive reaction for flavonoids was indicated by a yellow-orange colour change (Mir *et al*. 2016).

Alkaloids were tested with three reagents, namely Meyer’s, Wagner’s and Dragendorff’s reagents, following procedures described in Mir *et al*. (2016). To begin, approximately 1 mL of ethanol extract was mixed with 3 mL of chloroform and 3 drops of ammonia. The chloroform fraction was then separated and acidified with 10 drops of 2M sulfuric acid (H_2_SO_4_). Following this, each acid fraction was tested with Meyer’s, Wagner’s and Dragendorff’s reagents, and the presence of alkaloids was confirmed by white, brown and red precipitations, respectively (Mir *et al*. 2016).

Tannins were detected by mixing approximately 1 mL of the crude Beneng taro leaves extract with ferric chloride (FeCl_3_) reagent in a test tube. A positive reaction was indicated by a colour change to blue-green (Mir *et al*. 2016).

The presence of steroids and terpenoids was tested by mixing 1 mL of the ethanol extract with acetic anhydride until fully submerged in a test tube, followed by heating for 5 min. After cooling, 1 drop of concentrated sulfuric acid was added along the side of the test tube. A brown ring formed between the two liquid layers the presence of steroids and triterpenoids. A green colour in the upper layer confirmed steroids, while a red color in the lower layer confirmed triterpenoids (Lewis 2006).

Phenolics compounds were identified by mixing 1 mL of the filtered extract with 2 drops of FeCl_3_ solution. A positive reaction was observed as a green or blue-green colour (Sembiring *et al*. 2018).

The total flavonoids content was determined using a spectrophotometric method with aluminum chloride (AlCl_3_) as a reagent. A stock solution of 1,000 μg/mL quercetin in ethanol was prepared and diluted to concentrations of 20 μg/mL, 40 μg/mL, 60 μg/mL, 80 μg/mL and 100 μg/mL. The sample extract solution was also prepared at a concentration of 1,000 μg/mL. For each standard and sample, about 0.5 mL was mixed with 1.5 mL ethanol, 0.1 mL of 10% AlCl_3_, 0.1 mL of 1M sodium acetate and 2.8 mL of distilled water. Following 30 min of incubation, absorbance was measured at 425 nm (Sembiring *et al*. 2018).

The total phenolics content was determined using a spectrophotometric method with the Folin-Ciocalteu reagent. A stock solution of 1,000 μg/mL gallic acid in ethanol was prepared and diluted to concentrations of 20 μg/mL, 40 μg/mL, 60 μg/mL, 80 μg/mL and 100 μg/mL. The sample extract was further prepared at a concentration of 1,000 μg/mL. For each standard and sample, 0.5 mL was mixed with 2.5 mL of Folin-Ciocalteu reagent and 2 mL of 7.5% Na_2_CO_3_. After 30 min of incubation, absorbance was measured at 734 nm (Nooin *et al*. 2020).

### Antioxidant Activity Test

Approximately 0.4 mM DPPH solution was prepared as a free radical reagent. Ascorbic acid was used as a positive control with a concentration series of 1 μg/mL, 2 μg/mL, 3 μg/mL, 4 μg/mL, 5 μg/mL and 6 μg/mL. A stock solution of 500 μg/mL was prepared at concentrations of 12.5 μg/mL, 25 μg/mL, 50 μg/mL, 75 μg/mL, 100 μg/mL and 125 μg/mL by dissolving 10 mg of sample extract in ethanol in a 20 mL volumetric flask. Each sample and control solution were mixed with 600 μL of DPPH and diluted with 3 mL of methanol. The mixture was vortexed and incubated in the dark for 30 min before absorbance was read at 512 nm using a UV-Vis spectrophotometer (Nooin *et al*. 2020).

#### Identification of active compounds by LC-MS

LC-MS/MS analysis was performed using a UPLC-QToF-MS system (Waters, USA) equipped with an electrospray ionisation (ESI) source operating in positive mode. Ion source parameters were set as follows: capillary voltage 3.0 kV, cone voltage 30 V, source temperature 120°C, desolvation temperature 350°C, desolvation gas flow 800 L/h. Mass accuracy was maintained at ≤ 5 ppm using a lock-mass reference (leucine enkephalin, m/z 556.2771). Calibration was performed daily with sodium formate solution. Chromatographic separation used a C-18 column (2.1 × 100 mm, 1.7 μm) with mobile phase A (water + 0.1 % formic acid) and B (acetonitrile + 0.1 % formic acid) under a gradient elution at 0.2 mL/min. The extract (500 mg) was dissolved in 50 mL methanol, and 5 μL was injected into the system. Polar compounds eluted first, followed by less polar compounds. Peaks were analysed using MassLynx 4.1 software, and tentative identifications were made by matching mass spectra with reference database (Widiastuti *et al*. 2023).

### *In Silico* Analysis

Drug-likeness screening was performed on the bioactive compounds from Beneng taro leaves to assess whether each compound possessed drug-like properties. This screening was conducted using the SwissADME webserver (http://www.swissadme.ch/). The bioactive compounds that met the drug-likeness criteria were then subjected to molecular docking targeting the KRAS protein (P01116). Sotorasib (CID: 137278711), which was a well-known KRAS inhibitor, was used as the control drug. Molecular docking was carried out using PyRx 0.8 software (https://pyrx.sourceforge.io/), and the results were visualised through BIOVIA Discovery Studio (https://www.3ds.com) (Hidayatullah *et al*. 2022; 2023).

## RESULTS

Plant samples were collected from three different areas in Bogor Regency and City: TB-1, TB-2 and TB-3. Data from the Bogor Regency Central Statistics Agency (2021) showed that the TB-1 area, located in Kopo Village, Cisarua Sub-district, had an altitude of 802 m–855 m above sea level (masl). The daytime temperature here ranged between 18°C and 25°C, dropping to 10°C to 15°C at night, with humidity levels of 70%–90%. The region also experienced high light intensity on clear days. TB-2, located in Menteng, Bogor Barat Sub-district, sat at an altitude of 300 masl–350 masl. This area had a daytime temperature range of 22°C to 30°C, potentially dropping to 20°C at night, with about 80% humidity (Bogor City Central Statistics Agency 2021). Lastly, TB-3, at 210 masl–260 masl, had average daytime temperatures of 23°C to 27.5°C, and humidity levels were approximately 78% (Sudiar *et al*. 2019).

**FIGURE 1:**
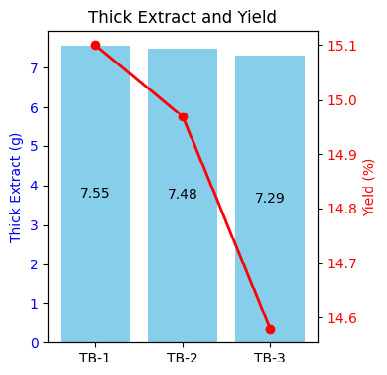
Beneng taro leaves extraction properties from different locations.

The moisture content of Beneng taro leaves simplicia was determined to be 15.18% for TB-1, 5.89% for TB-2 and 5.53% for TB-3. Additionally, the moisture content of the ethanol extract was 13.79% for TB-1, 15.48% for TB-2 and 13.53% for TB-3 (Fig. 1). The extraction yield and the phytochemical screening of secondary metabolites showed variation across locations, indicating environmental impact on metabolites biosynthesis.

**FIGURE 2:**
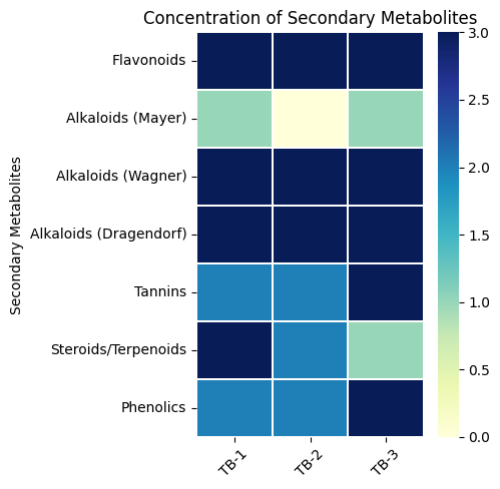
Phytochemical screening results. The colour corresponds to the concentration of secondary metabolites: 0 = no colour change, 1 = slight colour change, 2 = moderate colour change, 3 = intense colour change.

TB-1 had high concentrations of flavonoids, alkaloids and steroids/terpenoids, with moderate levels of phenolics and tannins. Meanwhile, TB-3 contained the highest concentration of phenolics and tannins, which might contribute to its stronger antioxidant activity. TB-2 showed relatively lower concentrations of several metabolites, particularly phenolics and steroids/terpenoids, which could explain its intermediate antioxidant activity (Fig. 2). The variations in secondary metabolite profiles across these samples highlighted the impact of growing locations on the phytochemical composition and potential bioactivity of Beneng taro leaves.

The highest levels of phenolics and flavonoids compounds were observed in the TB-1 sample, with a total flavonoids content of 94.48 ± 1.61 mg QE/g and phenolics content of 97.35 ± 1.74 mg GAE/g. In comparison, TB-2 and TB-3 showed lower levels of these metabolites, as detailed in Table 1.

The antioxidant activity test showed that the extract with the lowest IC_50_ value was obtained from the TB-1 sample, measuring 42.96 μg/mL. In comparison, the TB-2 and TB-3 samples had higher IC_50_ values, with average readings of 81.19 μg/mL and 71.85 μg/mL, respectively. The antioxidant activity of the three samples from different locations showed significant differences (*p* < 0.05). Therefore, it could be concluded that the extract from the TB-1 had the best antioxidant activity among Beneng taro leaves extract (Table 2).

**FIGURE 3:**
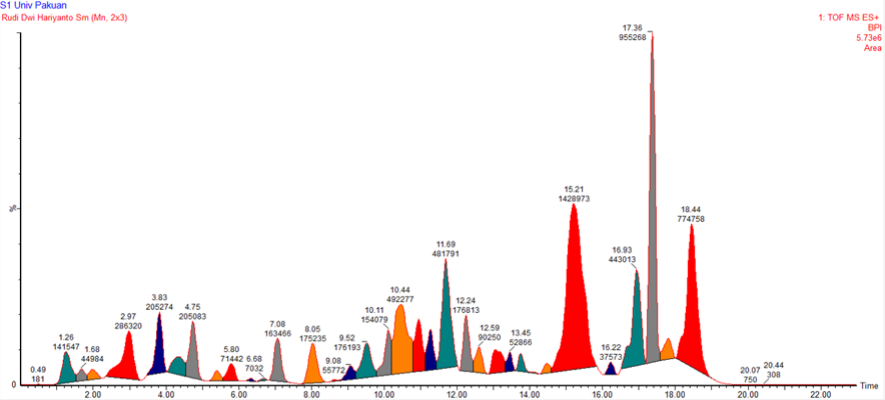
High-resolution LC-MS/MS chromatogram of Beneng taro leaf extract (TB-1) obtained with UPLC-QToF-MS. Peaks are annotated with retention times, and m/z values for each identified metabolite.

The secondary metabolites of the sample with the highest antioxidant activity (TB-1) were evaluated. LC-MS/MS analysis identified 16 metabolites in TB-1, particularly flavonoids such as quercetin, isorhamnetin, cochliophilin A, cyclovalone, hispidulin and oleocanthal (Fig. 3).

**FIGURE 4:**
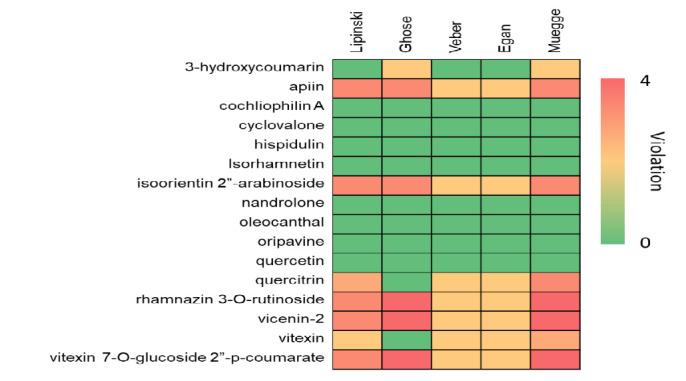
Drug-likeness screening of *Xanthosoma undipes* K. Koch compounds based on Lipinski, Ghose, Veber, Egan and Muegge rules. The figure now includes a legend defining each rule and colour-coding compounds that pass all criteria.

Drug-likeness screening played a crucial role in drug discovery and development. By selecting bioactive compounds with desirable drug-like properties, this process aimed to identify more promising candidates, thereby accelerating drug development and reducing costs (Jia *et al*. 2020). In this study, drug-likeness screening was performed on bioactive compounds from Beneng taro leaves. Out of 16 identified compounds, 8 had drug-like properties, including cochliophilin A, cyclovalone, hispidulin, isoharmnetin, quercetin, oripavine, oleocanthal and nandrolone (Fig. 4).

**FIGURE 5:**
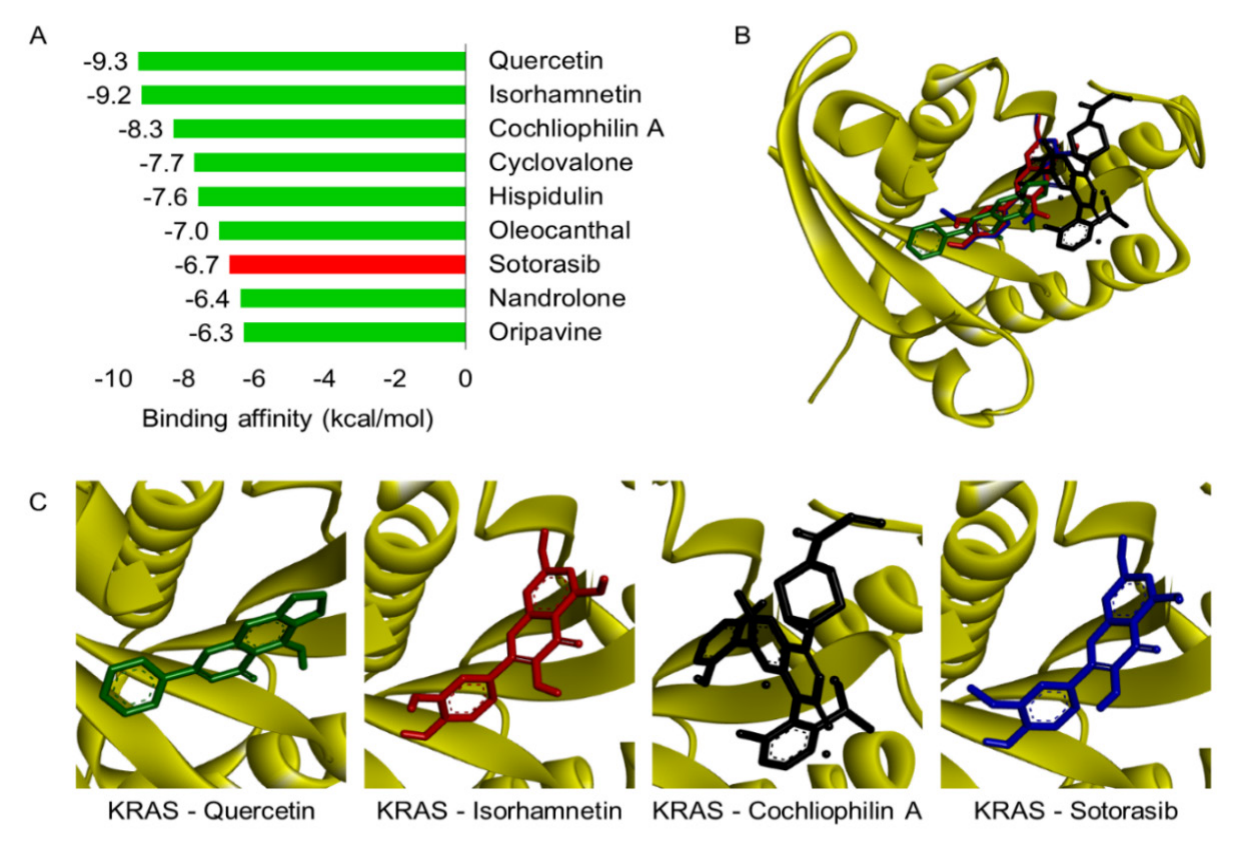
Molecular docking results. (A) Binding affinity scores of lead compounds against KRAS protein (values in kcal/mol); (B) Superimposition of the top three compounds with KRAS, highlighting binding pockets; (C) High-resolution 3D visualisation of quercetin, isorhamnetin, cochliophilin A and Sotorasib in KRAS active site, with hydrogen bonds, hydrophobic contacts and π–π stacking labelled.

**FIGURE 6:**
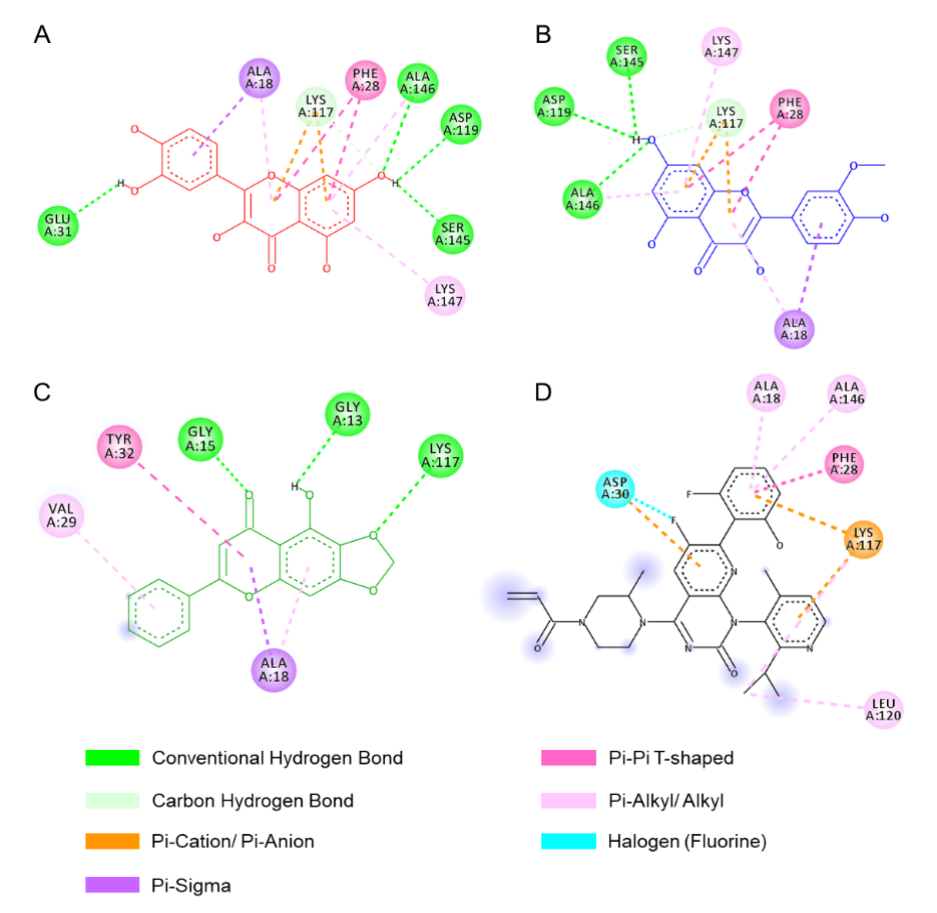
2D interaction diagrams between KRAS and each ligand: (A) Quercetin, (B) Isorhamnetin, (C) Cochliophilin A and (D) Sotorasib (Key amino acid residues, hydrogen bonds [green dashed lines] and hydrophobic interactions [pink arcs]).

The selected compounds were then subjected to molecular docking analysis targeting the KRAS protein, which played a critical role in cancer progression (Yang *et al*. 2024). Drug-likeness screening showed that eight compounds met drug-like properties. Additionally, molecular docking analysis showed that quercetin, isorhamnetin, and cochliophilin A had the strongest binding affinity against KRAS, a key cancer target (Figs. 5 and 6).

## DISCUSSION

The preparation of Beneng taro leaf extract began with the production of simplicia powder to facilitate maceration. Moisture content was determined as a key quality parameter, as excessive moisture can promote microbial growth and degrade active compounds. The moisture content of simplicia was 15.18% for TB-1, 5.89% for TB-2 and 5.53% for TB-3. These values complied with the maximum limit of 10% for traditional herbal raw materials set by the Indonesian Food and Drug Authority (Ismaniar *et al*. 2024), ensuring suitability for extraction. Maceration with 96% ethanol (1:10 ratio) was used to extract secondary metabolites while minimising degradation. The resulting macerate was concentrated under reduced pressure (40°C–55°C) to yield a dark green extract. Moisture content of the ethanol extracts ranged from 13.53% to 15.48%, within acceptable limits for preserving bioactivity.

The analysis results showed that the highest levels of phenolics and flavonoids compounds were observed in samples from the TB-1. The TB-1 represented a highland area characterised by relatively lower temperatures compared to the other two locations (Table 1). Numerous investigations have reported that plants responded to both high and low temperatures by modifying the synthesis of flavonoids and phenolics in species-specific ways. Generally, high temperatures could inhibit biosynthesis and cause flavonoids degradation. Although flavonoids accumulation in cold conditions relied on light exposure, several investigations indicated that lower temperatures supported flavonoids production with higher hydroxylation levels (Jaakola & Hohtola 2010). The role of altitude in secondary metabolites production have been extensively discussed, as plants adapt their metabolic pathways to counteract environmental stressors such as UV radiation, lower temperatures, and oxygen levels (Oleszek 2002). Furthermore, exposure to varying environmental factors, including soil nutrients and humidity played a crucial role in secondary metabolites biosynthesis (Shamloo *et al*. 2017).

A strong correlation was observed between phenolics and flavonoids content and antioxidant activity. The lower IC_50_ value for TB-1 supported the hypothesis that increased secondary metabolites concentration enhanced free radical scavenging ability. These results were consistent with previous reports that phenolics-rich plant extract had higher antioxidant potential due to its ability to donate hydrogen atoms and neutralise reactive oxygen species (Cosme *et al*. 2020).

The metabolites profiling of Beneng taro leaves extract from TB-1 group, which had the best antioxidant activity in this study, was conducted using LC-MS/MS. Based on the interpretation of LC-MS/MS data, 16 metabolites were successfully identified, primarily belonging to the flavonoids group. The compounds detected in the TB-1 extract included oleocanthal, cyclovalone, vicenin-2, isoorientin 2″-arabinoside, apiin, quercitrin, vitexin, vitexin 7-O-glucoside 2″-p-coumarate, cochliophilin A, quercetin, isoharmnetin, hispidulin, rhamnazin 3-O-rutinoside, 3-hydroxycoumarin, nandrolone and oripavine.

This study showed that the majority of Beneng taro leaves bioactive compounds demonstrated a more favourable binding affinity than the control drug, known as Sotorasib. Among these compounds, cochliophilin A, cyclovalone, hispidulin, isoharmnetin, quercetin and oleocanthal, demonstrated more favourable predicted binding affinities to KRAS than the control drug Sotorasib. Importantly, the top three compounds bound to the same active site as Sotorasib, suggesting potential relevance for KRAS-targeted therapies.

However, it must be emphasised that these docking results are *in silico* predictions and do not confirm biological efficacy. Experimental validation through *in vitro* cytotoxicity assays and *in vivo* studies is essential before drawing conclusions about anticancer activity. Furthermore, the development of these compounds into functional foods or pharmaceuticals will require consideration of factors such as bioavailability, stability during processing, safety profiles and regulatory approval pathways. While the presence of multiple bioactive compounds with favourable pharmacokinetic properties is promising, successful translation into consumer products will depend on meeting these practical and regulatory requirements.

Overall, the findings indicate that optimising Beneng taro cultivation in high-altitude regions could enhance bioactive compound yield. Future research should focus on compound isolation, biological validation of antioxidant and anticancer activities, assessment of bioavailability in human models, and evaluation of stability under different processing conditions. Integrating these studies with cost–benefit and feasibility analyses will be critical for realising the potential of Beneng taro leaves in functional food and pharmaceutical applications.

## CONCLUSION

In conclusion, planting location significantly influenced Beneng taro leaf phytochemistry. TB-1 (high altitude) yielded the highest flavonoid (94.48 ± 1.61 mg QE/g) and phenolic (97.35 ± 1.74 mg GAE/g) levels, with the strongest antioxidant activity (IC_50_ = 42.96 μg/mL, *p* < 0.001). LC-MS/MS profiling identified 16 metabolites, with quercetin, isorhamnetin and cochliophilin A emerging as lead compounds based on molecular docking results. These compounds exhibited binding affinities to KRAS of −9.3 kcal/mol, −8.7 kcal/mol, and −8.5 kcal/mol, respectively, outperforming the reference KRAS inhibitor Sotorasib (−8.1 kcal/mol). Other active metabolites included cyclovalone, hispidulin and oleocanthal, all of which also demonstrated favourable binding scores. While these *in silico* results suggest promising KRAS inhibitory potential, such predictions require validation through cytotoxicity assays against relevant KRAS-mutant cancer cell lines, followed by *in vivo* efficacy and safety studies. Future research should prioritise the isolation and structural confirmation of the top-ranking compounds (quercetin, isorhamnetin and cochliophilin A), evaluation of their bioavailability and stability in various formulations, and exploration of their potential in functional foods or pharmaceutical products. Preclinical and clinical studies will be essential to substantiate the health claims and move toward potential therapeutic applications

## Figures and Tables

**TABLE 1:** Flavonoids and phenolics levels in samples from different locations.

Grown area	Flavonoids (mgQE/g extract)	Phenolics (mgGAE/g extract)	*p*-value (Flavonoids) vs. TB-1	*p*-value (Phenolics) vs. TB-1
Cisarua (TB-1)	94.48 ± 1.61^c^	97.35 ± 1.74^b^	-	-
Cimanggu (TB-2)	28.62 ± 0.71^a^	66.43 ± 1.40^a^	*p* < 0.001	*p* < 0.001
Bogor (TB-3)	59.53 ± 0.75^b^	79.42 ± 0.93^b^	*p* < 0.01	*p* < 0.01

*Note:* Different superscript letters indicate significant differences at the 5% level (Duncan’s test).

**TABLE 2:** Antioxidant activity of extract from different locations.

Grown area	IC_50_ (ppm)	*p*-value vs. TB-1
Cisarua (TB-1)	42.96^a^	-
Cimanggu (TB-2)	81.19^c^	*p* < 0.001
Bogor (TB-3)	71.85^b^	*p* < 0.01

*Note:* Similar letter notation indicates no significant difference at the 5% level according to Duncan’s test.
